# Measuring user experience to forecast use & sustainability of behavior change interventions: Theory and data from the Trial for Lunch is in the Bag

**DOI:** 10.1186/1748-5908-10-S1-A83

**Published:** 2015-08-20

**Authors:** Cindy Roberts-Gray, Courtney Byrd-Williams, Nalini Ranjit, Deanna Holescher, Margaret E Briley, Sara Sweitzer, Maria Jose Romo-Palafox

**Affiliations:** 1 Third Coast R&D, Inc, Galveston, TX, 77550, USA; 2 Michael & Susan Dell Center for Healthy Living University of Texas School of Public Health Austin Regional Campus, Austin, TX 78701, USA; 3 School of Human Ecology, The University of Texas at Austin, Austin, TX 78712, USA

## 

Process evaluation in the efficacy trial for Lunch is in the Bag examined utility of measuring user experience of the intervention to forecast use and sustainability. The multi-component multi-level behavior change intervention was implemented in Early Care and Education Centers (ECEC) to increase parents' packing of fruits, vegetables, and whole grains in their preschool children's sack lunches. Reach of the cluster-randomized trial was to 30 ECEC in Texas (intervention (I)=15, control (C)=15) with study enrollment of 200 ECEC staff (I = 119, C = 81) and 633 parent-child dyads (I = 351, C = 282). Time by treatment modeling showed the intervention increased packing of vegetables and whole grains. Implementation process was assessed in three phases. Phase I assessed readiness to launch the trial. The research team simulated user experience of the intervention and forecast use. Their median estimates ranged from 20% of parents doing the goal setting assignments presented in the newsletters to 75% of teachers doing the classroom activities. Nearly all of the team indicated teacher training would increase implementing actions. Phase 2 was analyses of implementation observations, activity logs, and surveys. Multi-attribute Evaluation (MAE) showed prevalence of implementing actions at intervention ECECs was consistent with the research team's forecasts. Only one ECEC was assessed "ready to sustain." Phase 3 was post-trial interviews with implementers. Classroom was the only component for which sustained use was reported. Characteristics of initial implementation reported at control ECECs that received the intervention after the trial were comparable to reports at intervention ECECs. Debriefing interview with the research team identified options for adaptations and programmed support to prepare Lunch is in the Bag for broad dissemination. These results demonstrate opportunities for improving dissemination and implementation by integrating into the efficacy trial a progression of readiness assessments that measure user experience of the intervention's use, usefulness, and usability.

## Funding

R01CA149643-01 National Cancer Institute.

